# Identification of the egusi seed trait locus (eg) and its suppressor gene associated with the thin seed coat trait in watermelon

**DOI:** 10.3389/fpls.2023.1018975

**Published:** 2023-01-30

**Authors:** Na Li, Dan Zhou, Nannan Li, Shengnan Kong, Jianli Shang, Wanting Zhu, Jiming Wang, Shuangwu Ma

**Affiliations:** The Laboratory of Melon Crops, Zhengzhou Fruit Research Institute, Chinese Academy of Agricultural Sciences, Zhengzhou, Henan, China

**Keywords:** egusi seed, suppressor gene, marker-assisted selection, candidate gene, seed coat, watermelon

## Abstract

Egusi watermelon has a unique egusi seed type, which could be useful for breeding both edible seeds and edible flesh in watermelon. However, the genetic basis of the unique egusi seed type is not clear. In the present study, we first reported that at least two genes with inhibitory epistasis were responsible for the thin seed coat (unique egusi seed type) in watermelon. Inheritance analysis of five populations, including F_2_, BC, and BCF_2_, suggested that the thin seed coat trait was controlled by a suppressor gene together with the egusi seed locus (*eg*) in egusi watermelon. Based on high-throughput sequencing technology, two quantitative trait loci located on chromosome 1 and chromosome 6 were identified for the thin seed coat trait in watermelon. One of the loci, the *eg* locus on chromosome 6, was finely mapped to a genomic region of 15.7 kb, which contained only one candidate gene. Comparative transcriptome analysis highlighted differentially expressed genes involved in cellulose and lignin synthesis between watermelon genotypes varying in the thickness of the seed coat and provided several potential candidate genes for the thin seed coat trait. Taken together, our data suggest that at least two genes are complementarily involved in the thin seed coat trait and will be useful for cloning novel genes. The results presented here provide a new reference for uncovering egusi seed genetic mechanisms and valuable information for marker-assisted selection in seed coat breeding.

## Introduction

Watermelon is an economically important crop of the Cucurbitaceae family that is popular not only for its sweet edible flesh but also for its edible seeds, which provide a significant source of nutrition and income in parts of the world, including China, Israel, Iran, and Africa (Zhang and Jiang, 1990). Egusi watermelon (*Citrullus mucosospermus*) has a close relationship with dessert watermelon (*Citrullus lanatus*) and might be derived from the same ancestral population (Guo et al., 2019). Egusi watermelon has been domesticated for seed consumption and is nutritious with a high seed oil percentage and a high protein content (Gusmini et al., 2004; Jarret and Levy, 2012; Prothro et al., 2012). In addition, unique edible-seed watermelon germplasms, which are local cultivars in Northeast China, are also produced for seed consumption.

Usually, the flesh of egusi watermelon is bitter, hard, bland, white, and inedible (Oyolu, 1977). However, several egusi watermelon germplasms are important sources of disease resistance genes or alleles, such as resistance to *Phytophthora* fruit rot (Kousik et al., 2012), resistance to race 1 or 2W of powdery mildew (Davis et al., 2007; Tetteh et al., 2010), resistance to gummy stem blight (Gusmini et al., 2005), and resistance to zucchini yellow mosaic virus (Guner et al., 2019). Therefore, egusi watermelon has substantial potential utilization value in modern watermelon breeding.

**Figure 1:**
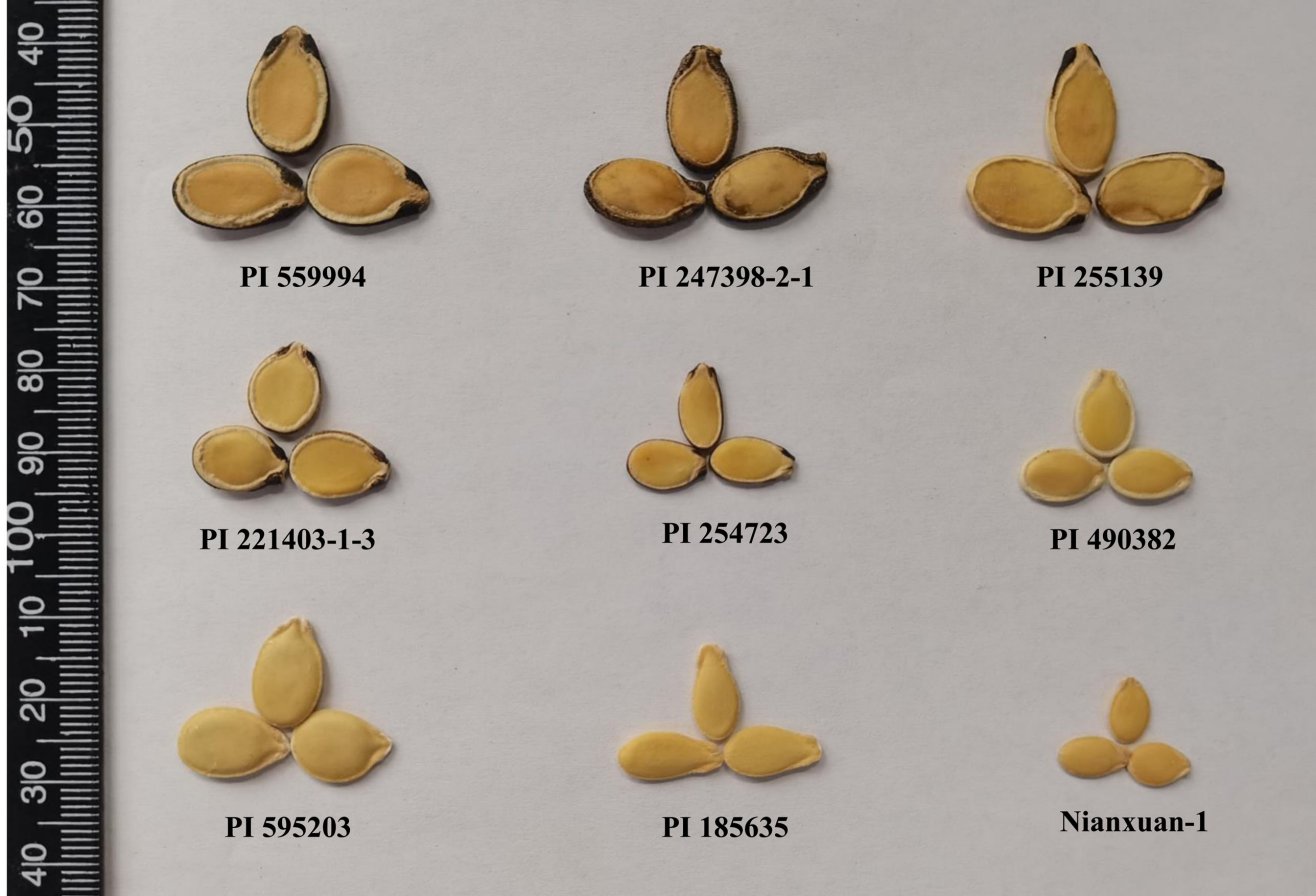
Different egusi seed types for egusi watermelon.

The seeds of dessert watermelon have a relatively hard and leathery seed coat, while the seeds of egusi watermelon have a thin membranous seed coat. Egusi watermelon possesses a unique seed trait in which the seed is enclosed in a fleshy pericarp at harvest, and the fleshy pericarp becomes a thin membrane after dying (Gusmini et al., 2004). The egusi seed type has been divided into different classes according to seed thickness, seed size, and seed edge thickness (Oyolu, 1977), but it has not been discussed whether all classes possess the egusi type seed coat. Based on the watermelon germplasms preserved in the National Mid-Term Genebank for Watermelon and Melon (Zhengzhou, China), egusi watermelon with a typical egusi type seed coat is shown in **Figure 1**. The type 1 class of seeds with thin seed coats and flat edges looks morphologically similar to the hull-less seeds in pumpkin (Lyu et al., 2022), which will be the focus of our research in the present study. The hull-less trait is an important trait in seed crops. The hull-less trait is usually controlled by a single or several genes. Both watermelon and pumpkin belong to the Cucurbitaceae family, so pumpkin is taken as an example: the hull-less pumpkin testa is the result of a single recessive gene with or without other modifying genes (Chahal et al., 2022; Lyu et al., 2022).

The egusi seed trait in watermelon has been analyzed mainly using several germplasms. It is common for egusi seeds to be controlled by a single recessive gene (*eg*) on chromosome 6 (Gusmini et al., 2004; Prothro et al., 2012; Luan et al., 2019; Paudel et al., 2019). The egusi seeds of most germplasms, including PI 490383w, PI 560006, PI 560023, PI 169233, and PI 186490, in these studies had thin seed coats and thick seed edges. However, the genetic basis of the totally thin seed coat type is not clear, and whether the inheritance of the unique egusi seeds in watermelon is associated with epistatic effects has not been explored.

Edible seeds of other cucurbits, such as pumpkin, squash, and bottle gourd (Idouraine et al., 1996; Chahal et al., 2022; Lyu et al., 2022), are also nutritious and form a part of the diet. Watermelon with edible egusi seeds and red, sweet, and edible flesh, like the soft seeds in pomegranate (Luo et al., 2020), would be extremely convenient for watermelon producers and consumers. Although attempts to breed edible flesh combined with egusi seeds have been made (Orji et al., 2016), progress had been relatively slow.

In the present study, we first revealed the genetic basis of the thin seed coat trait (unique egusi seed type) based on several independent and interrelated populations. Then, by integrating bulked segregant analysis sequencing (BSA-seq), restriction site-associated DNA sequencing (RAD-seq), RNA sequencing (RNA-seq), and fine-mapping tools, the chromosomal locations of the genes associated with the thin seed coat trait were identified. Based on the breeding goal of both edible seeds and edible flesh in watermelon, the main objectives of this study were as follows: 1) to identify the genetic basis of the thin seed coat trait (unique egusi seed type); 2) to identify the genomic regions related to the thin seed coat trait based on BSA-seq and RAD-seq; 3) to develop polymerase chain reaction (PCR)-based markers for marker-assisted breeding; 4) to fine-map the loci related to thin seed coats; and 5) to analyze potential candidate genes associated with thin seed coats based on RNA-seq.

## Materials and methods

### Plant materials, field experiments, and trait evaluation

A BC_1_F_1_ population (16CB1) of 461 individuals and an F_2_ population of 174 plants (19QF2) derived from a cross between B3 (P_1_, the female parent, *C. mucosospermus*) with typical egusi seeds (thin seed coat) and X1625 (P_2_, the male parent, *C. lanatus*) with thick black seed coat were constructed for further analysis. Some individuals from BC_1_ with egusi seeds were further selfed to create the BC_1_F_2_ population. One BC_1_F_2_ individual with thick yellow seed coats (non-egusi seed type) was selfing several generations and continuously selected as a male parent named B4; B4 was crossed with B3, and the resulting F_1_ plant was backcrossed with B3 to create another BC_1_F_1_ population (19CB2), which included 99 individuals.

All materials were obtained from the National Mid-Term Genebank for Watermelon and Melon (Zhengzhou, China). All parents and F_1_, F_2_, backcross BC_1_F_1_, and backcross BC_1_F_2_ populations were planted at the Zhengzhou Fruits Research Institute of the Chinese Academy of Agricultural Sciences in Zhengzhou. All parental, F_1_, and BC_1_F_2_ individuals were grown in triplicate with 10 plants each. The F_2_ and backcross BC_1_F_1_ populations were planted in a greenhouse following essentially regular management practices throughout the growing season. The fruits were harvested 45–50 days after manual pollination. Each fruit was cut lengthwise and immediately visually scored as the egusi or the non-egusi seed type. Pictures of typical fruit cross sections with seeds were taken. The seeds of each sample were investigated after drying to confirm the seed type phenotype.

Goodness-of-fit (χ^2^) tests were performed on the expected segregation ratios using SAS.

### RAD-seq and genotyping

The RAD protocol was employed as described previously (Baird et al., 2008). The enzymes and restriction fragment sizes were evaluated based on the “97103” reference genome sequence (ftp://cucurbitgenomics.org/pub/cucurbit/genome/watermelon/97103/v2/) (Guo et al., 2019). All libraries were sequenced using Illumina HiSeq sequencing PE150 platforms. For single-nucleotide polymorphism (SNP) and insertion/deletion (InDel) calling, the Burrows–Wheeler Aligner (BWA) (Li and Durbin, 2009) was applied for sequence alignment between the individual reads and the reference genome sequence, the Genome Analysis ToolKit (McKenna et al., 2010) was used to detect SNP loci, and SAMtools (Li et al., 2009) was used to filter out SNP loci. The filtering of SNP loci was based on three criteria: 1) an average sequence depth of <5-fold in the parents and <3-fold in the progeny; 2) no polymorphism between the parents; and 3) heterozygosity in the parents.

### Linkage map construction

The poorly performing markers were removed before map construction, which missed an excess of more than 50% of the missing data in the 19CB2 population. Markers with significant segregation distortion (χ^2^ test, P < 0.05) were excluded from the subsequent linkage map construction. The construction of the linkage map was performed using JoinMap 4.0 (http://www.kyazma.nl/index.php/mc.JoinMap) with a goodness-of-fit threshold of ≤5, a recombination frequency of <0.4, and a minimum logarithm of odds (LOD) score of 2.0. All genetic distances were expressed in centimorgans (cM), as determined by the Kosambi function (Kosambi, 2016). Linkage groups were assigned to chromosomes based on published high-density genetic map for watermelon (Shang et al., 2016).

### Gene mapping for the thin seed coat trait

Genome-wide quantitative trait locus (QTL) scanning was performed by adopting Bayesian model selection in the package R\qtlbim (www.qtlbim.org) (Yandell et al., 2007), which analyzes the QTL model for binary traits. The appropriate LOD threshold was determined by a permutation test of 1,000 repetitions (Churchill and Doerge, 1994). LOD scores corresponding to P = 0.001 were used to identify novel QTLs. The linkage maps and QTL results were visualized using MapChart software (Voorrips, 2002).

### Bulked segregant analysis sequencing (BSA-seq)

**Figure 2:**
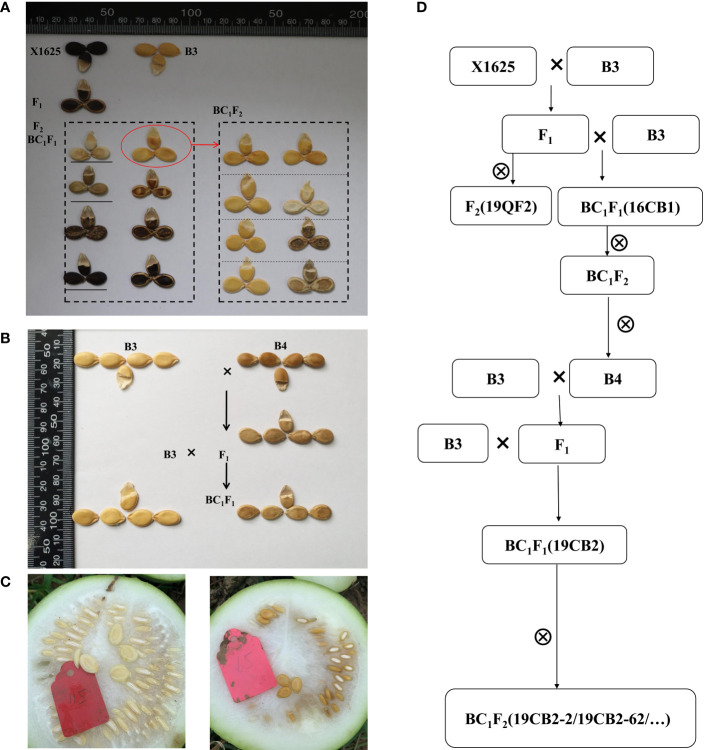
Seed types of the parents and progeny populations. **(A)** The seeds of B3, X1625, F_1_, F_2_, BC_1_F_1_, and partial BC_1_F_2_ individuals after drying. **(B)** The seeds of B3, B4, F_1_, and BC_1_F_1_ individuals after drying. **(C)** The fresh seeds of the egusi seed type (the same as B3, left) and non-egusi seed type (the same as B4, right). **(D)** The establishment process of the populations in the present study.

In addition to the egusi seed type, several non-egusi seed types were observed in the BC_1_F_1_ populations (16CB1) derived from B3 and X1625 (**Figure 2**). Plants with non-egusi seed types, including thick white seed coats, thick yellow seed coats, thick black seed coats, and thin yellow seed coats (unique egusi seed type), were thus selected, and DNA from each set of 20 plants was combined into three non-egusi pools (non-egusi1 pool, non-egusi2 pool, and non-egusi3 pool) and an egusi pool. The DNA of the pools was used for BSA-seq. All libraries were sequenced using the Illumina NovaSeq 6000 platform with PE150. BWA was applied following the method described previously (Takagi et al., 2013). A 250-kb sliding window with a 5-kb step size was employed for calculating the SNP index of the offspring pools, and Δ(SNP-index) was calculated as with the difference between the SNP indexes of the two offspring pools. The threshold at the confidence level of 99% was determined by 1,000 permutations for each window. Peaks that exceeded the threshold were considered as candidate QTL regions, and the candidate genes harbored in these regions were identified according to their annotation and SNP/InDel mutation.

### Genome-wide identification of SNPs and InDels and development of PCR-based markers

All libraries were sequenced using the Illumina HiSeq sequencing PE150 platform. The paired-end reads from parental watermelon lines were aligned to the reference genome sequence (Guo et al., 2019) using BWA. Conversions of sequence alignment files were performed using SAMtools. SNP and InDel mining was performed using GATK.

The extraction of 500 bp before and after SNP/InDel loci was performed by a self-compiled script in Perl. To develop PCR-based cleaved amplified polymorphic sequences (CAPSs) and derived CAPS (dCAPS) markers, the web-based free software dCAP Finder 2.0 (http://helix.wustl.edu/dcaps/dcaps.html) was used to find appropriate restriction enzymes for detecting SNPs (Neff et al., 2002). Primer 5.0 (Clarke and Gorley, 2001) and Oligo 7 (Rychlik, 2007) were used to design the appropriate PCR primer sets.

### Genotyping of PCR-based markers

DNA from all materials and populations used in the present study was extracted from young leaves using the Hi-DNAsecure Plant Kit (Tiangen Biotech, Beijing, China) according to the manufacturer’s instructions. PCR was performed in 25-μl reaction volumes containing 12.5 μl of 2× Power Taq PCR MasterMix (Bioteke, Beijing, China) with 10-μM primer (each) and approximately 50 ng of genomic DNA as a template. Thermocycling was initiated at 94°C for 5 min, followed by 35 cycles of 94°C for 20 s, 55°C–60°C for 30 s, and 72°C for 30 s, with a final extension at 72°C for 5 min. The PCR products were separated on an 8% polyacrylamide gel and visualized by silver staining.

### RNA-seq analysis and identification of differentially expressed genes

Seeds of genotypes with egusi seeds and non-egusi seeds were collected from fruit at 14, 21, and 27 days post anthesis (dpa). The embryo was removed, and the entire seed coat was quickly frozen in liquid nitrogen and stored at -80°C. For RNA-seq, the seeds were collected from five different fruits at one developmental stage for each genotype. Each genotype had 2–4 biological replicates. After the final cDNA library was synthesized, all cDNA samples were sequenced on the Illumina HiSeq sequencing PE150 platform. Analysis of differentially expressed genes (DEGs) was performed using TopHat and Cufflinks software (Trapnell et al., 2012). In brief, reads were mapped to the watermelon reference genome (Guo et al., 2019) using TopHat. Mapped reads were assembled using Cufflinks (Trapnell et al., 2010). The assembled transcripts from independent biological replicates were included and compared using Cuffdiff (Trapnell et al., 2010). Genes with a fold change in fragments per kilobase per million mapped reads (FPKM) >1 (log2 level) and false discovery rate (FDR) <0.001 were selected as DEGs.

## Results

### The thin seed coat trait was controlled by a suppressor gene together with *eg* in egusi watermelon

To unravel the genetic basis of the egusi seed type in egusi watermelon, F_2_ (19QF2) and BC_1_F_1_ (16CB1) populations derived from B3 and X1625 were constructed. B3, the female parent of the F_2_ and BC_1_F_1_ segregating populations, had a typical egusi seed type, and its seeds had only a thin yellow membrane after cleaning and drying. The seeds of X1625 had a thick black seed coat. B3 was crossed with X1625, and the resulting F_1_ plant was selfed to obtain the F_2_ population and backcrossed to B3 to create the BC_1_F_1_ population. All of the F_1_ plants had non-egusi seed phenotypes. Unexpectedly, individuals with the egusi seed type in the 19QF2 and 16CB1 populations were the same as B3, but individuals with the non-egusi seed type had different phenotypes (**Figure 2**). The seed type of non-egusi and egusi seed types in 19QF2 segregated at 148:26, fitting a 13:3 ratio (χ^2^ = 1.6558, P = 0.1982, **Table 1**), which suggested the presence of two genes including a suppressor gene for the thin seed coat trait. The seed types in randomly selected 16CB1 population segregated at 52:47, which fit a 1:1 ratio (χ^2^ = 0.2525, P = 0.6153). In addition, five individuals in 16CB1 with the egusi seed genotype and egusi seed phenotype were selfed to obtain the BC_1_F_2_ population. Some BC_1_F_2_ lines showed only the egusi seed type, while others showed segregation for non-egusi and egusi seed phenotypes (**Figure 2**), which further confirmed the occurrence of a suppressor gene for the *eg* locus, and the suppressor gene was masked in the 16CB1 population. These results suggested that the suppressor gene was a recessive gene and had no suppression effect on the main gene in this population.

**Table 1:** Segregation ratio of plants with non-egusi and egusi seeds in different populations.

Population name	Parental lines	Population type	No. of plants			Expected ratio	χ²	P value
			Total plants	Non-egusi seed	Egusi seed			
19QF2	B3 × X1625	F_2_	174	148	26	13:3	1.6558	0.1982
16CB1	B3 × X1625	BC_1_F_1_	99	52	47	1:1	0.2525	0.6153
19CB2	B3 × B4	BC_1_F_1_	99	54	45	1:1	0.8182	0.3657
19CB2-2	B3 × B4	BC_1_F_2_	99	71	28	3:1	0.5690	0.4506
19CB2-62	B3 × B4	BC_1_F_2_	109	76	33	3:1	1.6177	0.2034

### BSA-seq identified QTLs for the thin seed coat trait

**Figure 3:**
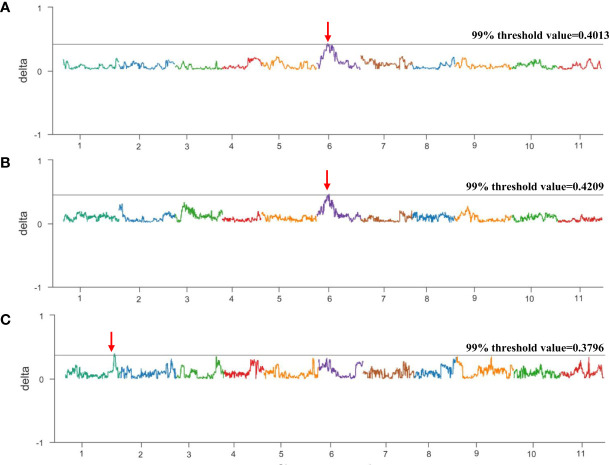
Bulked segregant analysis (BSA) of the BC_1_F_1_ population. **(A)** Δ (SNP index) graph from BSA-seq analysis between the egusi pool and non-egusi1 pool. **(B)** Δ (SNP-index) graph from BSA-seq analysis between egusi pool and non-egusi2 pool. **(C)** Δ (SNP-index) graph from BSA-seq analysis between egusi pool and non-egusi3 pool. The X-axis represents the chromosome, and the Y-axis represents Δ (SNP index). The dark gray line is the 99% threshold value; the region indicated by the red arrow is the putative QTL.

To map the genes related to the thin seed coat trait in egusi watermelon, three non-egusi seed types, including thick white seed coats, thick yellow seed coats, and thick black seed coats, and the egusi seed type (**Figure 2**) in BC_1_F_1_ (16CB1) were selected to create three non-egusi pools (non-egusi1 pool, non-egusi2 pool, and non-egusi3 pool) and an egusi pool. A total of 44 Gb of high-quality clean data were obtained by Illumina sequencing, and the average sequencing depth was 27-fold and more than 90% coverage for each (**Supplementary Table S1**). An average of 866,432 SNPs and 242,407 InDels were revealed for the pools. Using SNP index association algorithms, two regions on chromosome 6 of 6.055–7.095 Mb and on chromosome 1 of 32.695–33.420 Mb were found to exceed the threshold value at a 99% confidence interval level (**Figure 3**; **Table 2**).

**Table 2:** QTLs identified by BSA-seq.

	Chr.	From	To	Threshold at the 99% confidence level
egusi pool vs. non-egusi1 pool	Chr06	6,055,000	7,095,000	0.4013
egusi pool vs. non-egusi2 pool	Chr06	6,535,000	7,095,000	0.4209
egusi pool vs. non-egusi3 pool	Chr01	32,695,000	33,420,000	0.3796

### Identification of the main effect gene associated with the thin seed coat trait

The individuals with thick yellow seed coats in 16CB1 population were selfed to several generations and continuously selected, resulting in B4 with thick yellow seed coats. B4 was crossed with B3, and the resulting F_1_ plant was backcrossed to B3 to create another BC_1_F_1_ population (19CB2). All F_1_ progeny derived from B3 and B4 had non-egusi seed type, the same as B4, revealing recessive inheritance of the egusi seed type. In this study, 19CB2 segregated into 54 individuals with the non-egusi seed phenotype (the same as B4, **Figure 2**) and 45 individuals with the egusi seed phenotype (the same as B3, **Figure 2**), showing a ratio of 1:1 (χ^2^ = 0.8182, P = 0.3657, **Table 1**). The segregation analysis of the egusi seed type suggested that egusi seed production was controlled by one complete recessive gene in the 19CB2 population.

**Figure 4:**
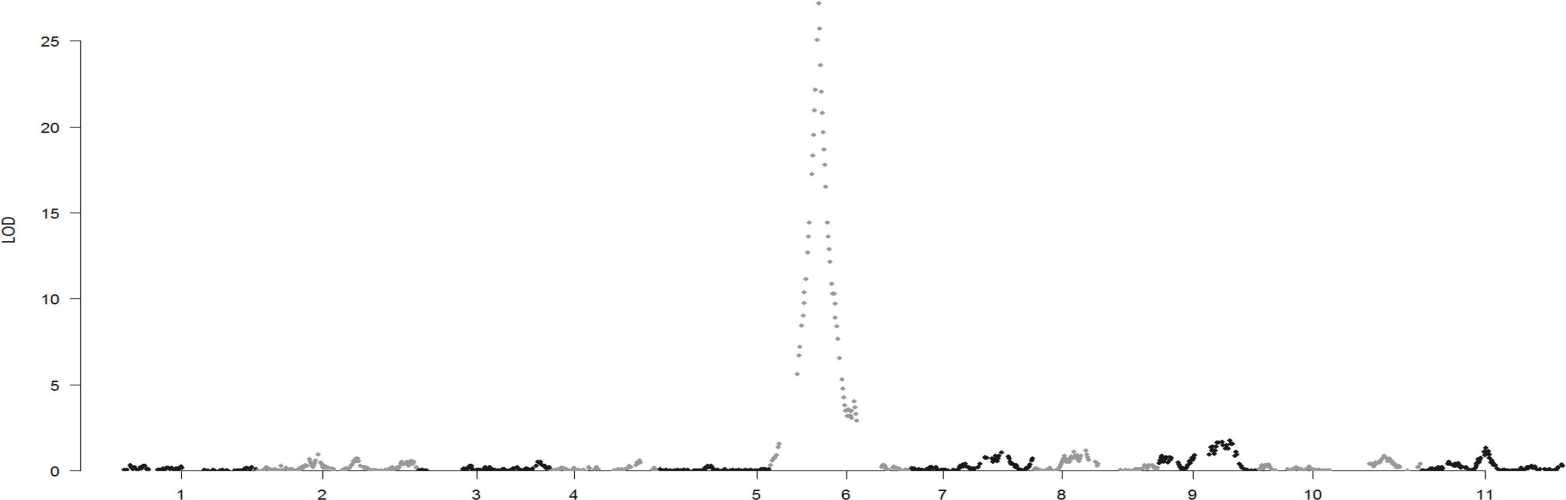
Genome-wide scan of QTLs for the thin seed coats in watermelon.

The genetic map, which contained 13,187 SNPs and 990 small InDels, was constructed (**Supplementary Table S2**) based on the RAD-seq data from B3, B4, and 99 19CB2 individuals. The total length of the linkage map was 903.8 cM, with an average distance of 0.06 cM between adjacent markers. The relationship between the genetic map and physical maps was mostly linear for each linkage group (**Supplementary Figure S1**). Genome-wide QTL analysis of the seed type trait was performed in the package R\qtlbim, which analyzes the QTL model for binary traits. One major QTL (*eg*), which explained 95.97% of the phenotypic variation and showed a peak LOD score of 27.2, was identified in linkage group 6 (**Figure 4**). We checked the genotype of the interval region of *eg* and found that only one individual (B2-28) had an inconsistent genotype and phenotype for all of the markers located in the QTL interval regions (LG6: 17.3–46.0 cM). The remaining 98 individuals in the 19CB2 population included 48 recombinant individuals and narrowed *eg* to a region of 452.7 kb from 6,910,159 bp to 7,362,904 bp on chromosome 6 (**Supplementary Table S3**).

### Fine-mapping of the main effect gene *eg* for the thin seed coat trait

To develop PCR-based markers for fine-mapping *eg* locus, three parental lines, B3, B4, and X1625 were resequenced using the Illumina platform. A total of 35 Gb of high-quality clean data were obtained, with an average sequencing depth of 32-fold (**Supplementary Table S1**). A total of 502,260 SNPs and 118,959 small InDels (InDel length 1–10 bp) were identified between B3 and B4. A total of 620,359 SNPs and 148,484 small InDels were identified between B3 and X1625 (**Supplementary Figure S2**). In the 452.7 kb QTL region, a total of 1,045 and 1,001 SNPs and small InDels were identified between B3 and X1625 and between B3 and B4, respectively. Of these genetic variants, 996 were common, and 161 and 115 were unique (**Supplementary Figure S3**). Based on resequencing data and BAM files of the parental lines, 12 InDels and 10 SNPs were developed into PCR-based markers to fine-map *eg* (**Table 3**). Nine InDel markers were genotyped in the 19CB2 population. InDel marker N6-45 found three recombinant individuals, while the other eight markers totally cosegregated with seed type phenotypes in all 99 individuals. The genotypes of all nine InDel markers showed that B2-28 had a consistent genotype and phenotype, which strongly supports the genomic region being associated with egusi seeds.

**Table 3:** The details for the PCR-based markers.

Marker type	Marker name	Chr.	Position (bp)	F-primer (5’–3’)	-primer (5’–3’)	Restriction enzyme
InDel	N6-45	Chr06	6,837,093	TTTTCTCCCTACCTTCCATT	AAAAGCAGAACAAAACGAAA	
InDel	N6-8	Chr06	6,924,191	AAACCGATACCAGGCAGAGT	AACCTACAGAGCGGGAACAT	
dCAPS	N6-9	Chr06	6,925,595	CATGAAACTCAAATCCGATT	TGTGGTGATCTGTTTTAACCTA	HinfI
InDel	N6-12	Chr06	6,927,920	ATGGTGTCTTTTCTTATGCT	TTTATCTATCGGGATGAGGG	
dCAPS	N6-13	Chr06	6,932,817	CATGCGTATTTACTTTCTCA	TTGTTATTGTTAAGGCCAG	EcoRII
InDel	N6-14	Chr06	6,949,700	AGCTTTAAGGGTCCACTTGATA	ATTTAGATTTTGGATGAACGGA	
InDel	N6-59	Chr06	6,952,704	AGCCAGAAACCGTGTAGATAAT	GGGAGCTGCACAAGACCAAGAA	
CAPS	N6-15	Chr06	6,957,443	TCCTGGAACAAGTGGAGATAAA	AGAAACCAAGCAATACCGTGAA	AluI
dCAPS	N6-17	Chr06	6,977,743	TTCTTGAACTTTCAATTGGC	ATGGCATCTAATAAGACACT	HaeIII
CAPS	N6-19	Chr06	6,993,638	CAAAGGGATACAATGTTAAA	GATGCTCAAGTTAGAAAATA	TaqI
dCAPS	N6-20	Chr06	7,004,917	CAATGGCAACGGGATTGTCG	ATAATTGTGGACGACTACCG	TaqI
dCAPS	N6-23	Chr06	7,038,080	AGTTTGGTGGACAGAGGAGA	CTTTCACTATACTATTCTT	MseI
dCAPS	N6-25	Chr06	7,040,484	GGAAATGAAAGTCTTGAGTAA	AATGAAATTGTTACTTCCA	NlaIII
dCAPS	N6-32	Chr06	7,046,144	TAATAGGGAAGAGTAGAGGG	TAAACCAAACACAAGCCAT	NlaIII
dCAPS	N6-35	Chr06	7,049,047	TATGAACATCAAGAATGAGCCA	AACCCTAAATATTCTACCA	EcoRII
InDel	N6-43	Chr06	7,056,134	ATTATCTTCAATGGCAAAACTG	CACTGTCTATCTATGAACCACG	
InDel	N6-44	Chr06	7,065,300	ATGTACTACCAATTTCGTGA	GTAGGTAATTTCAAATAAGTTCTA	
InDel	N6-50	Chr06	7,086,228	CCTATCCCTATCTTCAACCA	GACGATCCTCTTATTCCTCC	
InDel	N6-51	Chr06	7,112,888	TTCAGAAGCATAGTAGACCG	TTGGTTCAAGATTTTCGTTT	
InDel	N6-52	Chr06	7,208,182	TTCATAAATAATAGGAGGAGG	GGGATTGCATATTTGTTGTA	
InDel	N6-53	Chr06	7,247,400	GAAGTTCGGTCATGGTTTGG	AAATGGCTCAGTTGGTAGAA	
InDel	N6-54	Chr06	7,359,713	AATGTCGCATTATGCAAGTT	ATACCCATTTAACAAACAGC	

**Figure 5:**
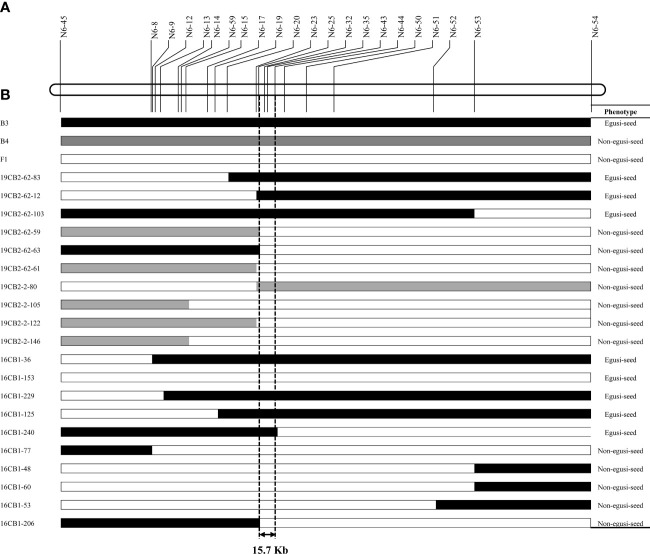
Graphical genotype of the recombinants and their seed type in the 19CB2 and 16CB1 populations. **(A)** Partial physical map of the genomic region of *eg* on chromosome 6. **(B)** *eg* was narrowed to a 15.7-kb region between N6-25 and N6-43 by analyzing the genotypes and phenotypes of these recombinants.

Two 19CB2 individuals, B2-2 and B2-62, with the heterozygous genotype for *eg* and non-egusi seed type, were selfed to obtain two BC_1_F_2_ populations. For the BC_1_F_2_ population derived from B2-2 and B2-62, 146 and 161 individuals were genotyped by three InDel markers (N6-14, N6-43, and N6-53), and 10 recombinant individuals were found. The seed types of 99 and 109 individuals from B2-2 and B2-62, respectively, were investigated. The non-egusi and egusi seed types in the BC_1_F_2_ derived from B2-2 and B2-62 segregated at 71:28 and 76:33, respectively, which both fit a 3:1 ratio (χ^2^ = 0.5690, P = 0.4506; χ^2^ = 1.6177, P = 0.2034). A total of 16 PCR-based markers were genotyped in 10 recombinant individuals and narrowed *eg* to a region of 209.3 kb between 7,038,080 and 7,247,400 bp (**Figure 5**). The phenotypes of BC_1_F_1_ and BC_1_F_2_ populations suggested that one egusi seed gene (*eg*) controlled the egusi seed type in this population.

A randomly selected BC_1_F_1_ population (16CB1) derived from B3 and B4 was firstly genotyped using six InDel markers, and five recombinant individuals were found. A total of four markers, namely, N6-59, N6-43, N6-44, and N6-50, totally cosegregated with the seed type phenotypes in all 99 individuals. N6-51, N6-52, and N6-53 were also genotyped in five recombinant individuals and finally narrowed *eg* to a genomic region between N6-45 and N6-53. Moreover, N6-59 and N6-43 were genotyped in another 362 individuals in the 16CB1 population, and five recombinants (16CB1-153, 16CB1-229, 16CB1-125, 16CB1-206, and 16CB1-240) were found. A total of 15 PCR-based markers were genotyped for these five recombinants. In this study, 16CB37-153 narrowed *eg* to a region upstream of N6-59, 16CB37-229 narrowed *eg* to a region upstream of N6-17, 16CB1-125 narrowed *eg* to a region upstream of N6-20, 16CB1-206 narrowed *eg* to a region upstream of N6-25, and 16CB1-240 narrowed *eg* to a region downstream of N6-43.

As a result, *eg* was narrowed to a 15.7-kb region (**Figure 5**) between N6-25 (chromosome 6: 7,040,484 bp) and N6-43 (chromosome 6: 7,056,134 bp), which contained only one putative gene (*Cla97C06G116000*) according to the watermelon genome annotation (Guo et al., 2019). Based on amino acid sequence analysis, *Cla97C06G116000* is predicted to encode *EPIDERMAL PATTERNING FACTOR-LIKE 4 (EPFL4)* protein (Takata et al., 2013). This protein is a member of the EPF/EPFL family, a group of cysteine-rich secreted peptides that regulate a range of developmental processes (Pillitteri et al., 2007; Sugano et al., 2010; Uchida et al., 2012; Bessho-Uehara et al., 2016; Kawamoto et al., 2020).

In addition, all 174 F_2_ individuals (19QF2) derived from B3 and X1625, the parents, and F_1_ were genotyped by eight PCR-based markers, namely, N6-45, N6-59, N6-32, N6-43, N6-50, N6-51, N6-52, and N6-53 (**Supplementary Table S4**). The results showed that all 26 individuals with the egusi seed type had the egusi seed genotype (B3 type). Of the 148 individuals with the non-egusi seed type, 17 lines had egusi seed genotype (B3 type) and 131 had non-egusi seed genotype (X1625 type or heterozygous). These results further confirmed that at least two genes controlled the egusi seed trait. These two genes might be located on different chromosomes and have a recessive epistatic relationship.

### Transcriptome profiles associated with cellulose and lignin synthesis in the egusi seed coat

The seed coat of the egusi seeds was soft until 27 dpa. The seed coat of the non-egusi seeds was soft until 21 dpa and became rigid at 27 dpa, which might be the result of lignification. Therefore, comparative transcriptome analysis between egusi seed and non-egusi seed genotypes at 21 and 27 dpa was performed. A total of 77.97 Gb of clean data with Q30 ≥92.58 and a G/C ratio ranging from 45.19% to 48.73% (**Supplementary Table S1**) were obtained. Then, differential gene expression analysis between egusi seed and non-egusi seed genotypes at different developmental stages was performed. A total of 671 DEGs were identified (**Supplementary Table S5**), of which 133 were commonly detected at the same developmental stages (**Supplementary Figure S4**).

**Figure 6:**
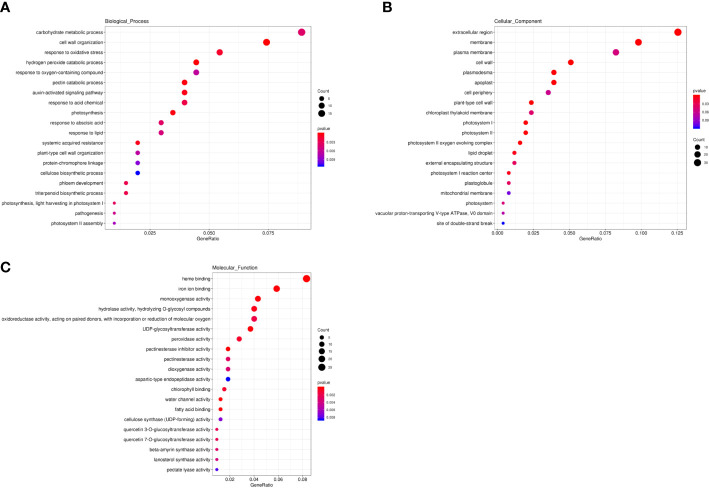
Functional enrichment analysis of DEGs. **(A)** Bar plot of GO terms involved in biological processes. **(B)** Bar plot of GO terms associated with cellular components. **(C)** Bar plot of GO terms involved in molecular functions.

In the genomic regions of chromosome 1 from 32,695,000 to 33,420,000 bp and chromosome 6 from 6,055,000 to 7,095,000 bp, only six genes were significantly differentially expressed in one specific data set (**Supplementary Table S6**). The expression level of *Cla97C06G116000* showed no significant difference in any data set. Moreover, a total of 2,887 SNPs and 975 small InDels (**Supplementary Table S7**) were identified in the genomic regions of these two loci. We checked variants within 3,000 bp upstream and downstream of the six DEGs and *Cla97C06G116000* and found 106 SNPs and 36 InDels between egusi and non-egusi genotypes. However, there were no obvious functional variants for these genes. In addition, 126 unique SNPs and InDels between B3 and X1625 in the region of chromosome 1 might be candidates for one of the loci associated with egusi seeds. These 671 DEGs were annotated against the Gene Ontology (GO) (Ashburner et al., 2000) database. GO enrichment analysis of these DEGs indicated that the associated GO terms mainly included carbohydrate metabolic process, cell wall organization, and pectin catabolic process in the biological process category (**Figure 6**); extracellular region and cell wall in the cellular component category (**Figure 6**); and heme binding, monooxygenase activity, UDP-glycosyltransferase activity, and cellulose synthase (UDP-forming) activity in the molecular function category (**Figure 6**). Several cellulose synthase genes (*Cla97C02G035420*, *Cla97C02G035440*, *Cla97C07G138170*, and *Cla97C10G194080*); lignin biosynthesis-related genes, which encoded glycosyltransferase or UDP-glycosyltransferase (*Cla97C02G033320*, *Cla97C04G070610*, *Cla97C07G130770*, *Cla97C08G150900*, *Cla97C08G150910*, *Cla97C09G174190*, *Cla97C09G174200*, *Cla97C09G176260*, *Cla97C09G176360*, *Cla97C09G179880*, *Cla97C10G202900*, and *Cla97C10G202960*); phloem development-associated genes (*Cla97C06G116210*, *Cla97C06G116240*, and *Cla97C06G116270*); and transcription factors involved in cellulose and lignin deposition, including auxin responsive protein (*Cla97C01G004700*, *Cla97C02G042420*, *Cla97C03G057790*, *Cla97C05G085700*, *Cla97C06G118630*, *Cla97C06G120580*, *Cla97C07G141380*, *Cla97C09G163980*, *Cla97C09G170150*, and *Cla97C11G210450*), MYB (*Cla97C01G018160*, *Cla97C02G026730*, *Cla97C04G071640*, *Cla97C08G148320*, *Cla97C09G179450*, and *Cla97C10G195200*), WRKY (*Cla97C01G021390* and *Cla97C04G074730*), NAC (*Cla97C10G198100*), and bZIP (*Cla97C07G135710*) domain gene families were differentially expressed between egusi and non-egusi seed genotypes (**Supplementary Table S5**), highlighting their roles in the regulation of seed coat formation in egusi seeds.

The egusi seed type is one of the typical characters of egusi watermelon, which might be involved in the domestication process from *C. mucosospermus* to *C. lanatus* landraces. Of the DEGs detected, 19 showed significantly different expression between egusi seed and non-egusi seed genotypes (**Supplementary Table S5**) and were located in domestication sweep regions (Guo et al., 2019).

## Discussion

Epistasis is a phenomenon in which the effect of one genetic variant is modified or masked by one or more other genetic variants and plays a critical role in quantitative genetic analysis (Van Steen, 2012). Previous classic genetic studies on egusi watermelon revealed a single recessive gene (*eg*) controlling the egusi seed trait (Gusmini et al., 2004; Ren et al., 2014; Luan et al., 2019; Paudel et al., 2019). The partial egusi seed type in watermelon (unique egusi seed type in the present study) is similar to the hull-less trait in seed crops. Previous studies showed that one major gene, a minor gene with modifiers, or a polygenic model controlled the hull-less trait in other seed crops, such as pumpkin (Chahal et al., 2022; Lyu et al., 2022), barley (Taketa et al., 2008), oil palm (Singh et al., 2013), and maize (Wang et al., 2005). In the present study, two genes with inhibitory epistasis were found to be responsible for egusi seeds in watermelon as evidenced by the F_2_ ratio (13:3) and confirmed by the phenotype and genotype in the BC_1_F_2_ population. In addition, BSA-seq also revealed that two genomic regions on different chromosomes associated with egusi seeds. For the seed coat type of egusi watermelon, several different classes were identified (**Figure 1**) in different germplasm resources, which also implies a complex genetic basis for the egusi seed type phenotype.

Previous studies mapped the *eg* locus underlying egusi seeds to an overlapping region on watermelon chromosome 6 (Prothro et al., 2012; Ren et al., 2014; Luan et al., 2019; Paudel et al., 2019). The *eg* locus was mapped to the 4.28-Mb genomic region between 6.75 and 11.03 Mb (NW0248325 and NW0250248) on chromosome 6 (Prothro et al., 2012; Ren et al., 2014). Recently, Luan et al. (2019) mapped the *eg* gene to a 130.7-kb region from 6.915 to 7.046 Mb on chromosome 6 and speculated that *Cla97C06G115870* was the candidate gene for eg (Luan et al., 2019). However, Paudel et al. (2019) refined the egusi locus to a 398.25-kb region from 6.958 to 7.356 Mb, which did not contain the predicted gene *Cla97C06G115870*. In the present study, combining BSA-seq, RAD-seq, and resequencing technologies, the *eg* locus on chromosome 6 was narrowed to a genomic region of 15.7 kb, which completely overlapped with that identified by Paudel et al. (2019). Five independent and interrelated populations, including 1,041 individuals/lines, were used to map and fine-map *eg* and confirm the occurrence of the suppressor gene. BSA-seq revealed two QTLs on chromosome 1 and chromosome 6 for the thin seed coat trait. According to the present study, the QTL on chromosome 6 was the common QTL (*eg*) for egusi seeds. However, whether the QTL on chromosome 1 is the suppressor gene needs further confirmation. Notably, a novel germplasm with egusi seeds could be obtained by using the *eg* locus and a smart design. Two dCAPS markers (N6-32 and N6-35) were the optimal choice for molecular marker-assisted breeding. Based on the molecular markers for seed size, flesh color, fruit shape, and resistance to zucchini yellow mosaic virus and Fusarium wilt (Ling et al., 2009; Li et al., 2017; Li et al., 2018; Li et al., 2020; Zhang et al., 2020; Li et al., 2021), by several generations of selection based on genotype and phenotype, novel germplasms (**Supplementary Figure S5**) with small hull-less egusi seeds, red flesh, a high total soluble sugar content (10%), and resistance to zucchini yellow mosaic virus and Fusarium wilt were obtained.

The hull-less trait is an important trait in seed crops. An ethylene response factor family gene regulating a lipid biosynthesis pathway controls the covered/naked caryopsis phenotype in barley (Taketa et al., 2008). A single gene, *NAC SECONDARY WALL THICKENING PROMOTING FACTOR1 (NST1)*, accounts for the hull-less trait in pumpkin (Lyu et al., 2022). Previous studies suggested that “naked-seed” pumpkin is the result of reduced cellulose and lignin contents in the hull-less seed coat at seed maturation (Bezold et al., 2005; Lyu et al., 2022). In the present study, comparative transcriptome analysis between egusi and non-egusi seed genotypes was performed to reveal the potential candidate genes and regulatory pathway involved in seed coat formation in egusi watermelon. Interestingly, several DEGs involved in cellulose and lignin biosynthesis were identified. Previous researchers found that monolignol glucosylation catalyzed by UDP-sugar glycosyltransferases (UGTs) was essential for normal cell wall lignification (Lin et al., 2016; Baldacci-Cresp et al., 2020). Twelve genes encoding glycosyltransferase or UDP-glycosyltransferase were significantly differentially expressed. Moreover, two DEGs, *Cla97C08G150900* and *Cla97C08G150910*, presented high expression in the non-egusi seed genotype but no expression in the egusi seed genotype, suggesting that these genes may play a key role in the formation of the seed coat in egusi watermelon. In addition, cellulose synthase (CES) may play an important role in the seed coat lignification (Sullivan et al., 2011; Griffiths et al., 2015; Yang et al., 2019). Four CES genes were significantly differentially expressed in the present study. Several transcription factors, such as NAC, WRKY, MYB, and bZIP (Zhong et al., 2007; Zhong et al., 2008; Wang et al., 2010; Wang et al., 2016; Kasirajan et al., 2018), are thought to be involved in cell wall biosynthesis and potentially play important roles in regulating cellulose and lignin biosynthesis. We found a total of 20 genes that showed significant differential expression between egusi and non-esusi seed genotypes, which might be potential candidate genes involved in the downstream regulation of egusi watermelon. In the expanding region of the *eg* locus, except for two genes (*Cla97C06G115490* and *Cla97C06G115630*) that showed significant differences between egusi and non-egusi seed genotypes at certain stages (**Supplementary Table S6**), other genes showed no significant differences, including *Cla97C06G115870* and *Cla97C06G116000*, which might indicate that the major *eg* locus did not control egusi seeds at the transcriptome level. The only gene located in the narrowed 15.7-kb region for the *eg* locus, *Cla97C06G116000*, is predicted to encode an *EPFL4* protein, which regulates a range of developmental processes in plants (Pillitteri et al., 2007; Sugano et al., 2010; Uchida et al., 2012; Bessho-Uehara et al., 2016; Kawamoto et al., 2020). Disruption of the function of the *REGULATOR OF AWN ELONGATION 2* (*RAE2*) protein by an (EPFL1) gene is required for the awnlessness phenotype in Asian rice (Bessho-Uehara et al., 2016). The authors also identified *SUBTILISIN-LIKE PROTEASE 1 (SLP1)* as a protease required for *RAE2* processing in the rice spikelet. In addition, 19 DEGs located in the domestication sweep regions from *C. mucosospermus* to *C. lanatus* landrace (Guo et al., 2019) were also potential candidates. Therefore, how these genes regulate the formation of the seed coat for egusi watermelon requires further research.

In conclusion, in the current study, we investigated the genetic basis of the thin seed coat trait (unique egusi seed type) in egusi watermelon. Two genes with inhibitory epistasis were found to regulate the thin seed coat trait in egusi watermelon. Using a combination of BSA-seq, RAD-seq, and resequencing, the *eg* locus was mapped to a genomic region of 15.7 kb, which contained only one candidate gene. The obtained PCR-based markers were the best choices for molecular marker-assisted breeding. Several DEGs were potential candidates for the regulation of seed coat formation in watermelon. Egusi seeds have a thin seed coat, which is our target for breeding sweet watermelon with soft seeds, like those in pomegranate. In the near future, innovative germplasms with small and edible seeds in the flesh of the fruit, similar to easily swallowed fruits like seedless watermelon, will be extremely convenient for watermelon producers and consumers.

## Data availability statement

The datasets presented in this study can be found in online repositories. The names of the repository/repositories and accession number(s) can be found below: CNSA (https://db.cngb.org/cnsa/) of CNGBdb with accession code CNP0003272.

## Author contributions

NL and SM contributed to the conception and design of the study; NL, NNL, SK, WZ, and JS performed the experiments. JS, SM, DZ, and JW contributed reagents/materials/tools. NL wrote the manuscript. All authors contributed to the article and approved the submitted version.
