# Supplementary figures and images for: Identification of the egusi seed trait locus (eg) and its suppressor gene associated with the thin seed coat trait in watermelon
Source: Front Plant Sci. 2023 Jan 30;14:1018975. doi: 10.3389/fpls.2023.1018975 (PMC9923051; doi:10.3389/fpls.2023.1018975)

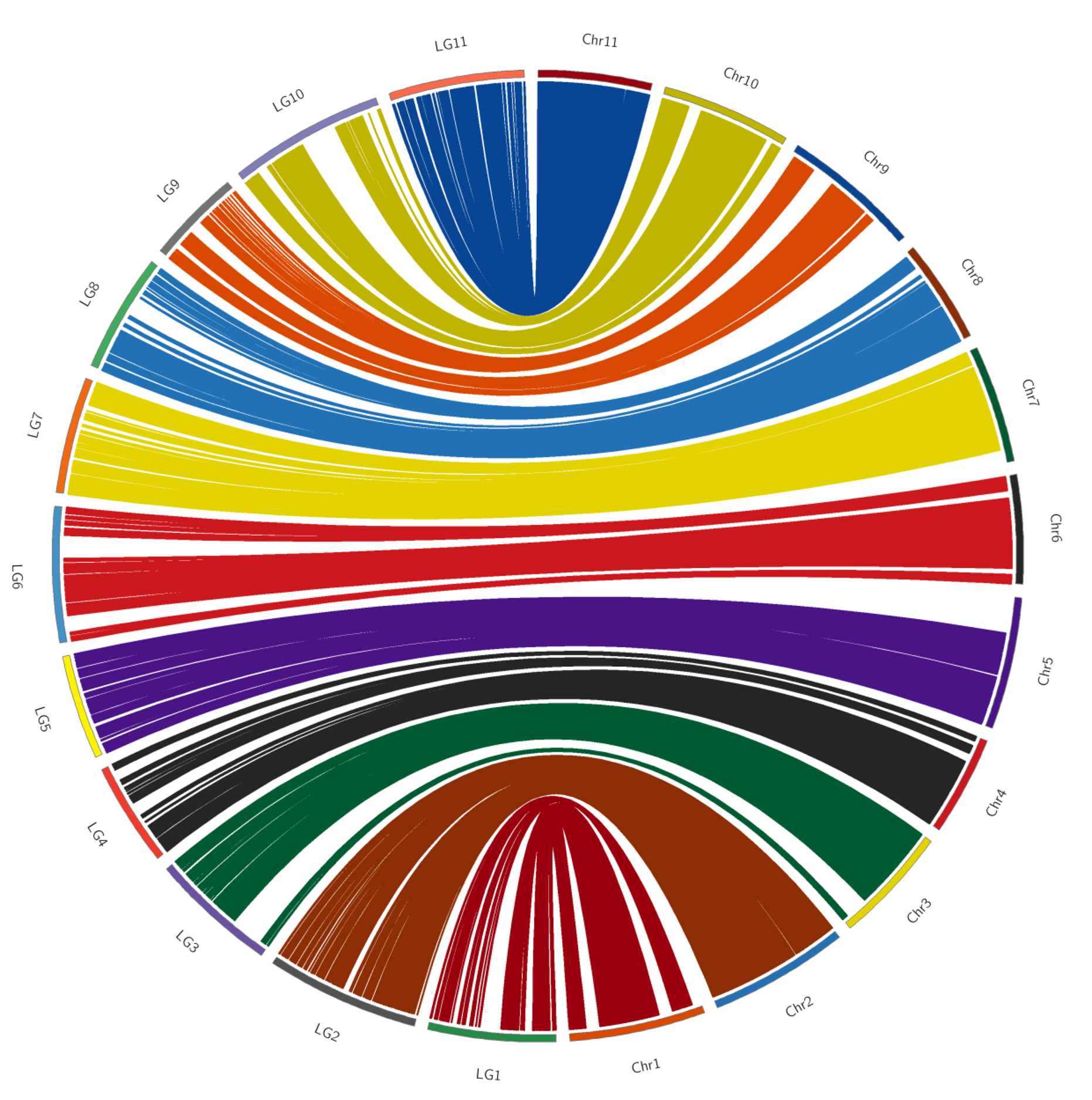

Supplement: Supplementary file 1 [file Image_1.jpeg]

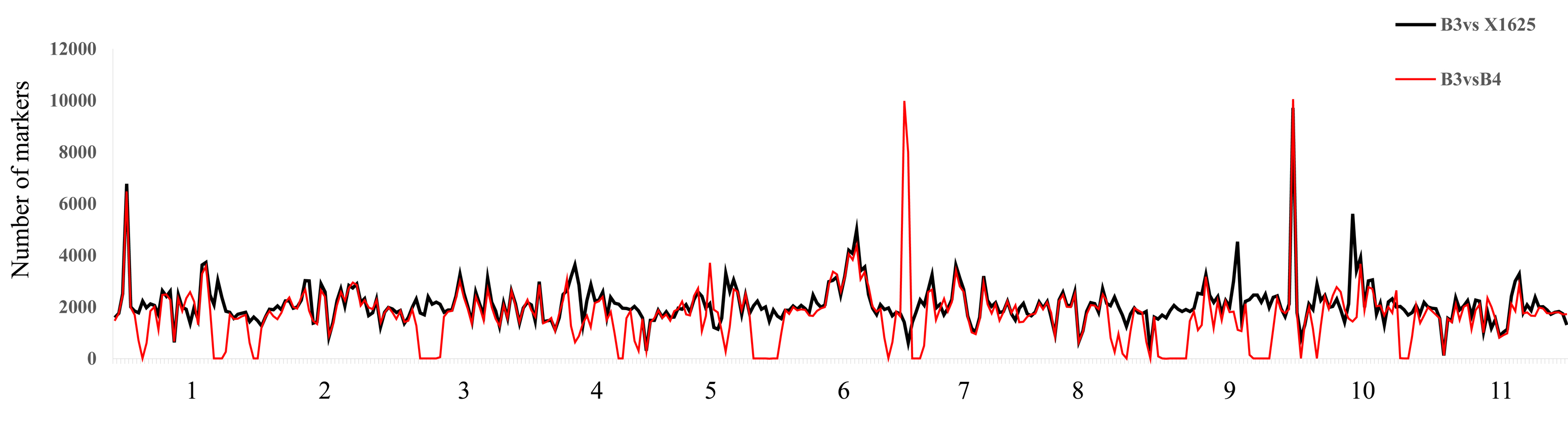

Supplement: Supplementary file 2 [file Image_2.jpeg]

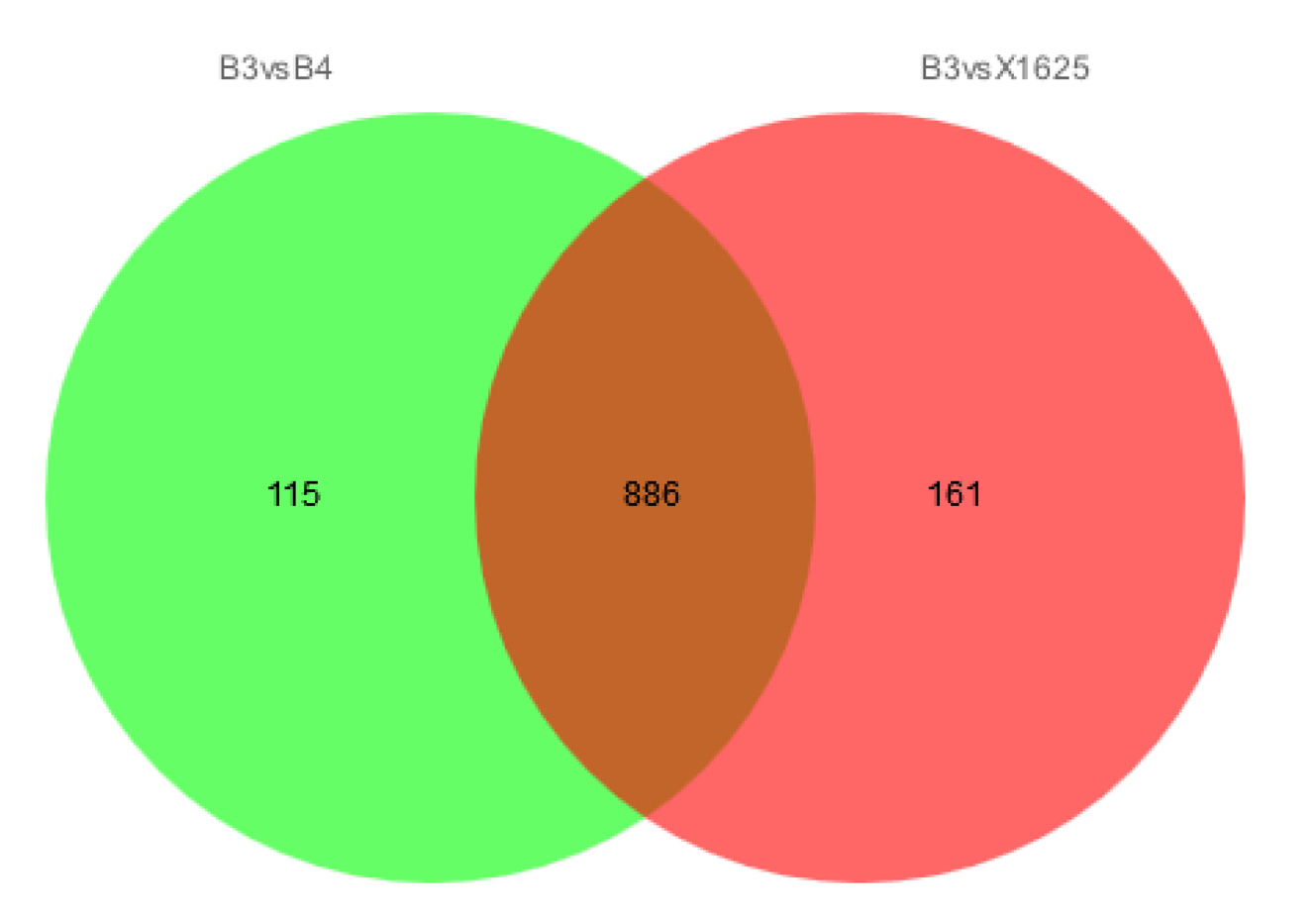

Supplement: Supplementary file 3 [file Image_3.jpeg]

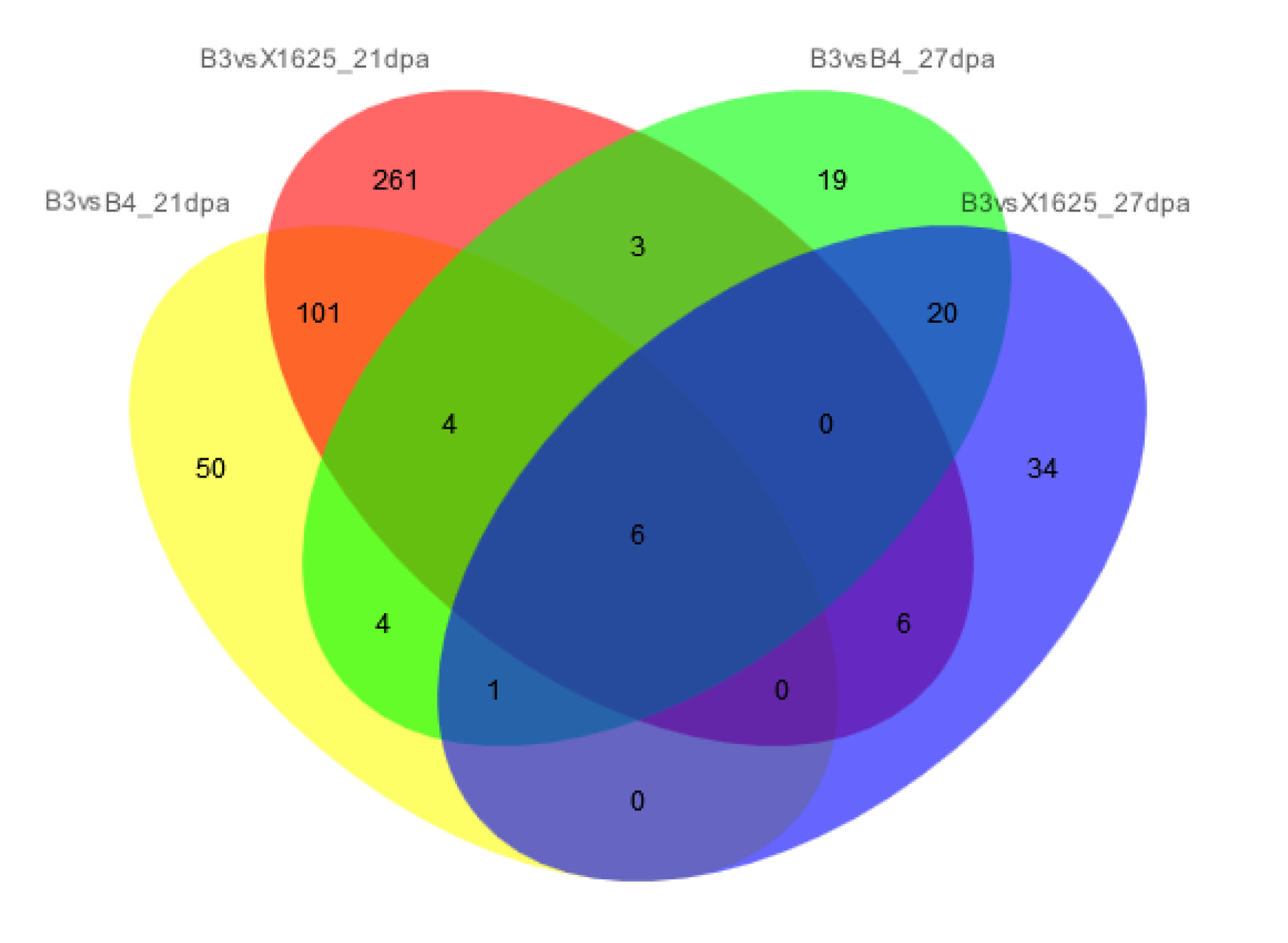

Supplement: Supplementary file 4 [file Image_4.jpeg]

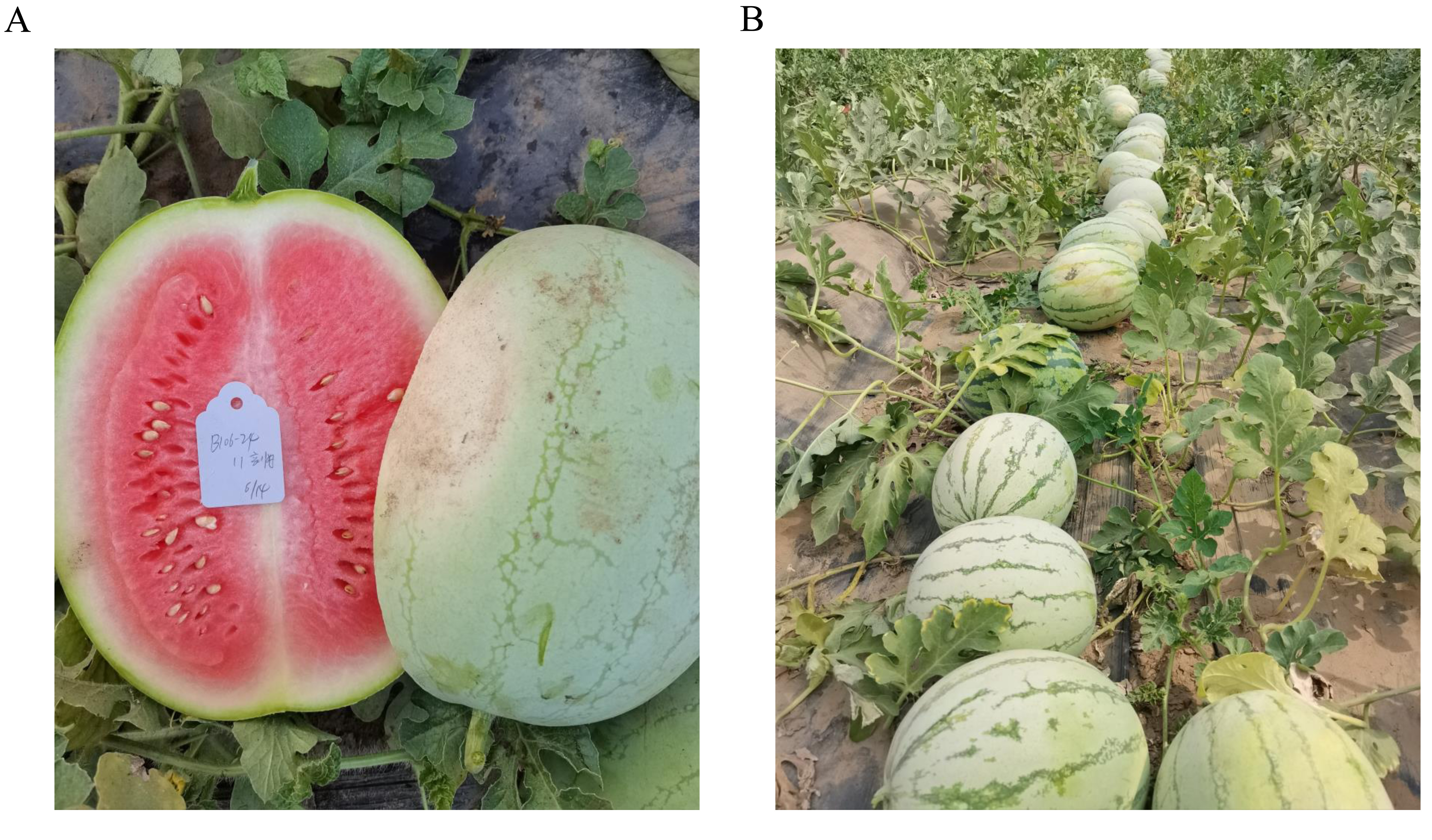

Supplement: Supplementary file 5 [file Image_5.jpeg]
